# Evaluation of an evidence‐based veterinary medicine exercise for instruction in clinical year of veterinary medicine program

**DOI:** 10.1002/vro2.3

**Published:** 2021-04-02

**Authors:** Philippa M. Gibbons, Stacy L. Anderson, Stanley Robertson, Faythe K. Thurman, Julie A. Hunt

**Affiliations:** ^1^ College of Veterinary Medicine Lincoln Memorial University Harrogate TN USA; ^2^ Health Sciences Library West Virginia University Morgantown WV USA

**Keywords:** clinical veterinary student, evidence‐based veterinary medicine, journal club

## Abstract

**Introduction:**

Evidence‐based veterinary medicine (EBVM) is a fundamental core competency for new graduates. Our objectives were to evaluate clinical students’ use, understanding of, and confidence in EBVM before undertaking an exercise consisting of an hour seminar in conjunction with a medical librarian, followed by a journal club and to evaluate students’ knowledge of and ability to apply EBVM following the exercise.

**Methods:**

In this cohort study, students undertaking a large animal ambulatory rotation completed questionnaires before the seminar, following the journal club, and at graduation. Students took a Fresno test evaluating EBVM knowledge at the end of the rotation.

**Results:**

Prior to the seminar, 94% of students defined EBVM at least somewhat accurately, and 51% reported EBVM was at least 'quite important'. During previous rotations, 71% of students had performed literature searches to answer a clinician‐assigned question; 89% had done this to answer their own questions. Students with previous research or EBVM training were more likely to perform self‐directed literature searches. The most frequently used resource was textbooks. Eighty‐seven per cent and 90% of students found the seminar and journal club, respectively, at least moderately useful in improving EBVM knowledge.

**Conclusions:**

Our results support the inclusion of an EBVM exercise during the clinical year and suggest spaced repetition may be helpful in teaching this topic.

**Abbreviations**
EBVM: evidence‐based veterinary medicine
LMU: Lincoln Memorial University
PBL: problem‐based learning

## INTRODUCTION

Evidence‐based veterinary medicine (EBVM) can be defined as 'decisions that combine clinical expertise, the most relevant and best available scientific evidence, patient circumstances and owners’ values'.^1^ EBVM comprises five steps: formulating a clinical question, searching the literature, critically appraising the evidence, applying the evidence and evaluating performance.^2^ The need to train new graduates in EBVM has never been greater, as technology presents access to an incredible amount of quickly available information including online textbooks and peer‐reviewed literature, boarded specialists via subscription veterinary websites and clinical advice from veterinary colleagues via social media. Veterinary clients have access to more information than ever before, which may prompt more frequent discussion of EBVM between client and veterinarian.

The American Association of Veterinary Medical Colleges has stated that all graduates should be able to identify, review and critically evaluate biomedical literature and apply it to the practice of contemporary EBVM. At a practical level, the students should be able to recognise the importance of EBVM and apply its principles by (1) identifying a knowledge gap, (2) formulating a relevant question to address the knowledge gap, (3) identifying and utilizing appropriate resources of scientific merit to address the knowledge gap, (4) critically appraising the relevant information and published evidence, and (5) integrating and applying new knowledge to the practice of veterinary medicine.^3^

But are veterinary practitioners using EBVM? Eighty‐six percent of veterinarians internationally reported having heard of EBVM; positive respondents were more likely to be clinicians, have worked in the UK or to have a post‐graduate qualification.^4^ A Belgian study found that veterinarians consulted colleagues, specialists, laboratories and the internet more frequently than peer‐reviewed publications which is not in keeping with EBVM principles.^5^ An international study found that clinicians were more likely to utilise the veterinary information network (VIN), an online community of veterinarians, when seeking information for cases, whereas non‐clinical veterinarians most frequently utilised PubMed, a peer‐reviewed literature database.^6^ In a 2015 UK survey, practitioners reported reading practical review magazines (*In Practice* and *Veterinary Times*) the most frequently among print sources, with Google being the most accessed electronic source;^7^ both of these sources rank lower on the pyramid of evidence.^1^ The pyramid of evidence is a visual representation of where sources rank in quality of evidence from general internet search at the bottom and systematic review at the top.^8^

Veterinarians have diverse reasons for not utilising EBVM. When compared to human medicine, veterinary practitioners possess a paucity of high quality evidence such as systematic reviews, meta‐analyses and equivalent of the Cochrane reviews. Private practitioners often must pay to access full‐text articles. Practitioners have expressed a dislike of EBVM's rigid focus on clinical trials which may not represent their local populations and feel that EBVM ignores the value of their clinical experience and expertise. Veterinarians also cited lack of time and training as reasons for not using EBVM.^9^ For EBVM to reach widespread usage among veterinarians, training in EBVM concepts is essential for veterinary students. In fact, most survey respondents reported hearing about EBVM in veterinary school, although literature and continuing education courses were also common responses.^4^ Teaching EBVM in veterinary school poses several challenges, including integrating the topic into a heavy curriculum and teaching clinical concepts in the clinical curriculum.^2^

Methods for teaching evidence‐based medicine (EBM) have been extensively researched in the human medical field. A systematic review of 12 randomised controlled trials of EBM teaching modalities evaluated a library‐based workshop, multidisciplinary or sole discipline 2‐day sessions, problem‐based learning (PBL), computer‐based versus lecture, directed workshops versus self‐directed computer‐based, librarian‐associated and didactic versus active sessions. Five studies investigated changes in competency, and all studies demonstrated improvement. Overall, didactic teaching was superior to PBL session, a librarian increased competency, and online learning to supplement work‐based experience was more effective.^10^ Another study showed that critical appraisal skills of residents were improved by utilising an EBM approach to journal club.^11^ As required of the veterinary core competencies, EBVM should be integrated throughout the curriculum. A longitudinal training program that included librarian‐led basic search skills in the first year, two didactic lectures and a 3‐hour workshop in the second year, with further exercises in the clinical year increased pharmacy students’ EBM knowledge and skills.^12^

In veterinary medical education, there are reports of EBVM being taught at various stages of the curriculum using a range of methods including lectures, assignments, demonstrations and student presentations.^13^ Other EBVM teaching methods reported in the literature include development of critically appraised topics for clinical year students using the first four steps of EBVM,^14^, ^15^ and first year students.^16^ An EBVM exercise that involved steps 1–3 of EBVM with a focus on EBVM of pharmacology was administered during a clinical anaesthesia rotation.^17^ Other clinical year students evaluated an exercise that required students to use a literature evaluation form to appraise one of two given articles on large animal reproduction.^18^ Although these studies describe and evaluate EBVM interventions, none of them report using a validated assessment of students’ knowledge and understanding of EBVM. This study adds to the literature by describing an exercise where the students completed the first four steps of EBVM in a clinical setting in a journal club along with a taught seminar. In addition, to the authors' knowledge, no studies of EBM in veterinary medicine currently utilise a validated assessment piece.

The objectives of this study were to evaluate students’ prior knowledge, use of, understanding and confidence in EBVM from prior research and exposure to EBVM before taking an EBVM seminar and journal club style exercise which taught EBVM steps 1–4 during a clinical rotation and to evaluate the seminar and exercise on assessing students’ knowledge and use of EBVM. We hypothesised that the journal club exercise would promote students’ EBVM knowledge, understanding and confidence both immediately after the exercise and at the time of students’ graduation.

## MATERIALS AND METHODS

All fourth year Lincoln Memorial University (LMU) veterinary students are required to complete the large animal ambulatory rotation at the DeBusk Veterinary Teaching Center, which includes a mandatory EBVM exercise. Students in the Class of 2019 previously were exposed to EBVM in the second year of the curriculum during the One Health course which involved researching a case study paper, performing critical analysis and recommending best practices with a public health emphasis. In addition, prior to starting clinical year, they had received an hour‐long seminar on how to use LMU's library website and its resources. On the rotation, the EBVM exercise consisted of a seminar on EBVM followed by a journal club exercise. The seminar was delivered by two of the authors, one a food animal clinician (Philippa M. Gibbons) who was the rotation course coordinator and the other a librarian (Faythe K. Thurman) who delivered the literature searching component. This exercise was introduced to ensure all students graduated with knowledge and understanding of EBVM and the ability to apply it to clinical cases, as well as address a known deficiency of critical thinking in the curriculum.

This study was reviewed and deemed exempt by the Institutional Review Board at LMU (human ethics approval). A convenience sample of 31 fourth year students was recruited from among the graduating class of 2019 and signed informed consent forms. The maximum group size on each rotation was 12 students, and the length of each rotation was 4 weeks. Data were collected from May 2019 to May 2020.

### EBM exercise

On the first day of the rotation, all students irrespective of whether they were enrolled in the study participated in an hour EBVM multimedia (Powerpoint) seminar given by the principle investigator (Philippa M. Gibbons) that covered the first four steps of EBVM (ask, acquire, appraise and apply) based on the Royal College of Veterinary Surgeons’ Knowledge Toolkit.^2^ The seminar presented an example of an EBVM exercise using the clinical question 'does oxytetracycline or procaine penicillin G result in faster resolution of bovine infectious keratoconjuctivitis lesions'. The literature searching and article retrieval steps were presented by the College of Veterinary Medicine's librarian (Faythe K. Thurman) and refreshed literature searching tools using LMU's Reed Health Sciences Library. Emphasis was placed on how participants would perform EBVM after graduation by informing them of different open access resources, and when EBVM should be employed in clinical practice. The design of the exercise was based on the four‐component instructional model.^19^

Following the seminar, all students on the rotation completed a journal club exercise in small self‐selected groups of two‐five students. Each group developed a unique clinical question that they wanted to answer based on a large animal case they had observed; defined the population, intervention, comparison, outcome (PICO) question; searched for and identified a single scientific paper on the topic; and presented the paper during a journal club in the third or fourth week of the rotation, depending on caseload and clinic schedule. The students used a worksheet (Appendix 1) and online resources of literature evaluation forms for different study types (https://casp-uk.net/) to assess the paper's validity, reliability and applicability for preparation of the journal club. As this was the first introduction to a journal club for the students, they were encouraged to utilise their written worksheet for presentation. During the journal club discussion, emphasis was made on the applicability of the paper to answer their clinical question. Multiple faculties were invited to the journal club session that lasted approximately 1 hour in total.

The final assignment of the EBVM exercise was a Fresno test of competence in EBM, which was adapted from a validated test used in human medicine but substituted clinical questions in food animal and equine medicine.^20^ The test was given as an assignment due on the last day of the rotation and was individual work. A rubric was used to grade students on the Fresno test (Appendix 3). The Fresno test was adapted from an online resource^21^. Together the journal club exercise and Fresno test accounted for 10% of the student's grade in the clinical rotation. A single author (Philippa M. Gibbons) graded all Fresno tests. The journal club exercise was graded using a rubric that included each step of the EBVM based on the worksheet and also students’ individual participation. As this was group work, the journal club grade was not included in the data collection for this investigation as not all members of the group were enrolled in the study.

### Questionnaire

Students enrolled in the study completed a pre‐seminar questionnaire that gathered details of demographics, previous EBVM and research experience and their confidence in the four steps of EBVM. The questionnaire was adapted from Johnston et al.^22^ Following the journal club, the students completed the second part of the questionnaire which asked their opinion of the usefulness of the seminar using a series of five‐point Likert scales. Finally, at the time of graduation from LMU, which was 1–11 months after their participation in the study, students completed the third part of the questionnaire which also used a series of five‐point Likert scales. This portion of the survey investigated to what extent they had utilised EBVM in subsequent clinical rotations. The full survey can be seen in Appendix 2.

### Data analysis

Results from the questionnaire were coded and entered into a spreadsheet where descriptive statistics were calculated. Continuous variables were described using mean and standard deviation (SD), while interval and ordinal data including Likert scale data were described using median and range. Likert scale data were compared using Mann Whitney U‐tests. Statistical testing was performed using Microsoft Excel and IBM SPSS Statistics version 26, and significance was set at p < 0.05.

## RESULTS

### Pre‐exercise questionnaire

Twenty‐nine (94%) of respondents were female, and two (6%) were male. Total, in the class of 2019, there were 82 females (80%) and 20 males (20%). Participants’ mean age was 27.3 (SD 4.0) with a range of 24–45 years. Three students (10%) had completed Master's degrees prior to veterinary school. Seventeen (55%) students reported that they had no research experience. Of the 14 (45%) that did report research experience, 13 (92%) had involvement in the data collection, with 11 (79%), nine (64%) and two (14%) having involvement in manuscript preparation, statistical analysis and project design, respectively. Class size for 2019 was 109 students. Students were not enrolled from 2/12 rotations due to delay in Institutional Review Board (IRB) approval and absence of the primary investigator (Philippa M. Gibbons).

When asked to define EBVM in the pre exercise questionnaire, 29 students (94%) made comments that fit within the definition of EBVM or listed one of the steps of EBVM. One student (3%) reported not knowing what it was, and one (3%) described it as making a differential list. Table 1 shows the themes that were reported by the students. Only eight students (26%) reported having previous teaching on EBVM. Of those, three (38%) had been taught EBVM during undergraduate course, three (38%) during professional (veterinary) school and two (25%) during veterinary school. Four (50%) of those reporting previous EBVM instruction received between 2 and 4 hours, 100% of which covered searching for an article, with 50% also covering article appraisal and 50% statistical interpretation. Only one student (3%) reported learning about development of a PICO question.

**TABLE 1:** Themes students used to describe their understanding of EBVM prior to the seminar and journal club exercise (only correct definitions included)

Theme	Number of students (%)
Statistics	3 (10.7)
True definition	16 (57.1)
Using peer reviewed literature	8 (28.6)
Study type	1 (3.6)

Twenty‐two students (71%) reported that on previous rotations, they had been asked to perform a literature search to answer a clinical question by a supervising clinician; 11 of them (50%) had done this two‐five times. Ten students (46%) reported taking 11–30 minutes to search, and four (20%) took over 60 minutes. Sixteen (73%) reported that they successfully downloaded an article, and 15 (65%) attempted to critically appraise the information they obtained. There was no significant difference between students that reported previous research experience or EBVM teaching and whether they attempted to appraise the information they obtained (p = 0.39 and 0.1, respectively).

Twenty‐five students (89%) reported that they undertook a literature search to answer a clinical question that was not asked of them, that is this was self‐directed learning. Fifteen of those students (60%) reported doing this two‐five times, with 12 (52%) spending 11–30 minutes on the search. Fourteen students (61%) did not attempt to appraise the article. There was no significant difference between students that reported previous research experience or EBVM teaching and whether they attempted to appraise the information obtained (p > 0.64 and 0.3, respectively). Students with previous research experience were more likely to perform self‐directed searches (p = 0.003) as were students with prior EBVM teaching (p < 0.001). Students in the first five rotations were more likely to perform both requested and self‐directed literature searches (p < 0.07 and 0.003, respectively).

**FIGURE 1:**
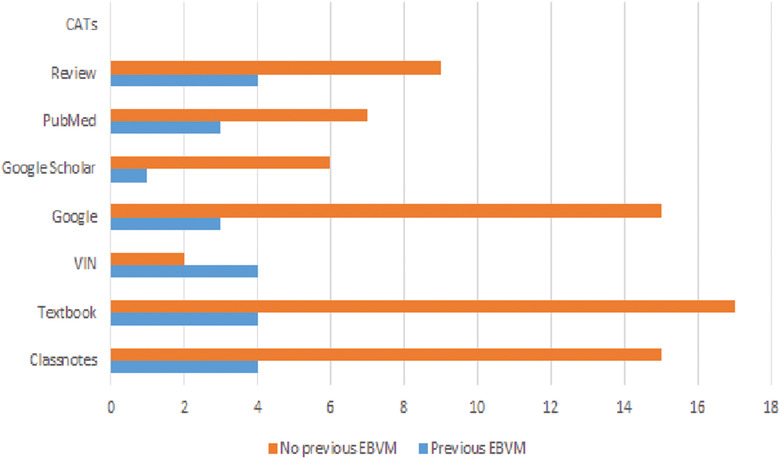
Utilization of each resource by students performing self‐directed research (n = 25) prior to the EBVM exercise, for students reporting whether or not they had previous EBVM training Abbreviations: CAT, critically appraised topic; VIN = veterinary information network.

Table 2 shows the percentage of students utilising each resource for either clinician‐led or self‐directed learning. There was no significant difference between resources used by students for requested or self‐directed literature searches (p = 0.97). Nineteen students (66%) reported using LMU's Reed Health Sciences Library to find articles and search databases. Figure 1 shows the number of times each resource was used for self‐directed research performed by students prior to the EBVM exercise between those reporting whether they had prior EBVM experience or not.

**TABLE 2:** Number of students utilising each resource for either clinician‐led or self‐directed learning prior to the EBVM exercise. Total number of students undertaking self‐directed was 24 and 22 undertaking clinician led literature searching

	Self‐directed	Clinician‐led
Class notes/blackboard	19	15
Text book	21	19
VIN	16	17
Google general	18	14
Google scholar	7	6
Pubmed/CAB abstract	10	16
Review articles	10	9
Critically appraised topics	0	0

The majority of students (16–51%) considered EBVM quite important. There was no significant difference in students’ responses between students with prior research or prior EBVM training. Table 3 shows the students’ reported confidence in performing each of the steps of EBVM at the start of the study via percentage of students.

**TABLE 3:** Students’ confidence in performing each of the steps of EBVM prior to the seminar and journal club exercise

	Formulating a clinical question n (%)	Find evidence n (%)	Search database n (%)	Evaluate clinical evidence n (%)	Use library resources n (%)	Identifying study type n (%)
Not at all confident	3 (9.7)	0	5 (16.1)	2 (6.5)	0	6 (19.6)
Slightly confident	14 (45.2)	8 (25.8)	12 (38.7)	17 (54.8)	5 (16.1)	11 (35.5)
Moderately confident	13 (41.9)	14 (45.1)	11 (35.5)	10 (32.2)	17 (54.8)	11 (35.5)
Quite confident	1 (3.2)	9 (29.0)	3 (9.7)	2 (6.5)	0	3 (9.7)
Extremely confident	0	0	0	0	0	0

### Post‐exercise questionnaire

Table 4 shows the results from the Likert scale questions on students’ thoughts on completion of the EBVM seminar, librarian seminar, Fresno test and journal club exercise on their improvement of EBVM knowledge. The majority of students reported that they were quite or extremely likely to use the information in future rotations (18–58.1%). There was no statistical significant difference in students’ responses between those with research experience and those without, those that had previous experience of EBVM or those in early or late rotations.

**TABLE 4:** Post‐exercise questionnaire

	Seminar in improving EBVM knowledge n (%)	Librarian seminar in improving searching n (%)	Librarian seminar and retrieving articles n (%)	Journal club exercise in improving EBVM knowledge n (%)	FRESNO test in solidifying knowledge n (%)
Not at all useful	1 (3.2)	3 (9.7)	1 (3.2)	0	2 (6.4)
Slightly useful	3 (9.7)	5 (16.1)	9 (29.0)	3 (9.7)	7 (22.6)
Moderately useful	14 (45.1)	10 (32.3)	11 (35.4)	15 (48.4)	12 (38.7)
Quite useful	12 (38.7)	11 (35.4)	9 (29.0)	12 (38.7)	9 (29.0)
Extremely useful	1 (3.2)	2 (6.4)	1 (3.2)	1 (3.2)	0

### Fresno results

Mean percentage grade on the Fresno test was 72.1% (SD 12.6), with a range from 53.7% to 92.6%. There was no significant difference in Fresno grade between those students with or without previous research experience, and those with or without previous EBVM experience or whether they undertook the exercise in the first or last five rotations.

### Questionnaire at graduation

Six of 31 students (19%) completed the exercise and study during the last rotation, and one student (3%) did not complete the questionnaire at graduation. This left 24 students who had rotations after the EBVM exercise on the large animal ambulatory rotation. Among them, 17 (83%) reported that they did not perform EBVM or develop a PICO question in subsequent rotations. Five students (21%) reported that they shared knowledge learned during the EBVM exercise with veterinarians they interacted with at clinical rotations. Students that undertook the rotation in the first five rotations, reported a trend that they were more likely to perform literature searches requested by the supervising veterinarian (p = 0.07).

## DISCUSSION

This study was the first to evaluate an exercise that included steps 1–4 of EBVM including a journal club with a validated test of understanding of EBVM. The Fresno test has been validated to assess competency in evidence‐based practice.^20^, ^23^, ^24^ After training, students’ mean Fresno scores were moderately strong at 72%. This indicates students were competent at performing the first four steps of EBVM immediately following completion of the exercise. These scores are higher than reported in a study of medical students where the Fresno test was used^25^, ^26^. In this study, only one author graded the Fresno test The Fresno test demonstrated that most students could effectively write a PICO question, list sources and their advantages and disadvantages and describe how to search, but they struggled with evaluating the reliability and validity of studies and assessing the magnitude and significance of the results. Students with previous EBVM or research training did not have higher Fresno scores than those without prior experience, suggesting either that the present training was by itself adequate for all students to become proficient at EBVM regardless of prior experience, or that students with prior experience had forgotten their past training. There was a lack of students (only 26%) reporting previous EBVM training however, with only three reporting this was during professional school. This is in conflict with what is described by the LMU CVM curriculum map, which indicated EBVM being taught in the second year, which involved researching a case study paper, performing critical analysis and recommending best practices with a public health emphasis as well as a literature searching seminar before the start of clinical rotations. This discrepancy could be due to both of these teaching opportunities not being presented as EBVM specifically. It is unclear whether students did not recognize that professional school meant veterinary school, whether they did not recognize that these previous exercises were within the scope of EBVM training, or whether they simply forgot having performed those exercises. This finding provides support for a spiral curriculum, where topics are revisited with increasing levels of difficulty as the student progresses through a curriculum; this spaced repetition is known to improve students’ memory of a topic.^27^ In addition, continued clarification of the definition of EBVM may be important.

Over half of participating students reported that EBVM was important. In the pre‐study survey, approximately 70% reported having performed a literature search to answer a clinical question when asked by a supervising clinician, and approximately 90% had performed a search of their own volition. Students with previous research experience or EBVM training were more likely to have performed self‐directed literature searches. Upon completion of the journal club exercise, 58% reported they were likely to use the information learnt in future rotations. However, despite the students’ favorable reports, in the 1–11 months between their participation and graduation, 83% did not perform EBVM or develop a PICO question in subsequent rotations. This may be partially explained by there being more students enrolled in the study in the second half of the clinical year, so they had fewer months to practice their EBVM skills. Further follow‐up would be interesting to see if the students continued to use principles learnt during the exercise in the first year post graduation.

Prior to the exercise, students were least confident in identifying a study type and most confident in finding evidence. The most common resources used by students to answer clinical questions were textbooks, followed by VIN for clinician‐led inquiries and class notes for self‐directed reviews, respectively. This was similar but not identical to the sources reportedly used by other veterinary students to develop critically appraised topics (CATs), which were primarily CAB Abstract, Medline and VIN,^16^ and similar to private practice veterinarians internationally, who utilised VIN, International Veterinary Information Service and PubMed.^6^ Students consulting sources for pharmacology rounds utilised different sources, including VetPrep, the Texas A&M library website, Google searches and PubMed.^17^

Most students reported that the librarian seminar, the clinician seminar and the journal club exercise were at least moderately useful in improving their EBVM knowledge. In another study of veterinary students, CAT development was considered an effective exercise (16, 28), and in medical residents, an evidence‐based approach to journal club improved EBM knowledge.^28^ Arlt and Heuwieser found that the literature evaluation form, similar to the one used in this study, was well received by students.^18^ Fewer students reported that the Fresno test was useful in solidifying their knowledge, but this is not surprising, since it was intended for assessment more than teaching. Most students also reported that the librarian was helpful for assistance in retrieving articles, suggesting that the librarian played an integral role in supporting their knowledge gain. A study by Koufogiannakis et al likewise found that human medical and dental students who were in the librarian‐associated PBL group had greater improvement in EBM knowledge.^29^ Based on our study results, during the subsequent clinical year, we maintained the EBVM exercise in the same form, with the exception of eliminating the Fresno test.

Limitations of this study included the small number of students (n = 31) that participated. Also, in retrospect, to more fully evaluate the ability of the exercise to improving student knowledge, understanding and ability to apply that knowledge, ideally two Fresno tests could have been delivered, one at the start of the rotation and one at the end. However, this may have further reduced the number of volunteer participants. Finally, grades from the journal club exercise were not able to be included in the data analysis because students worked in pairs, and both individuals were not necessarily enrolled in the study.

## CONCLUSIONS

In conclusion, an EBVM exercise that emphasised the applicability of EBVM in a clinical scenario, by developing clinical question and undertaking EBVM steps 1–4 (using development of a clinical question, PICO question, searching the literature to find the best article to answer the question and appraising the article) to answer that question, presented in a journal club style was well received by students. This exercise with inclusion of the journal club represents a novel form of including EBVM training to clinical students. The results also support the importance of a librarian's involvement in teaching EBVM. The moderately strong Fresno scores indicated that this exercise was effective in educating students on EBVM. The lack of students reporting previous EBVM training despite it being present in the curriculum suggests that spaced repetition may be helpful in teaching this topic to veterinary students. Students with previous EBVM and research experience however were more likely to perform self‐directed searches, indicating that EBVM training can be effective in the long term.

## AUTHOR CONTRIBUTIONS

Project design: Philippa M. Gibbons, Julie A. Hunt, Stanley Robertson and Stacy L. Anderson. Data collection: Philippa M. Gibbons and Faythe K. Thurman. Analysis and interpretation of data: Philippa M. Gibbons. Write up: Philippa M. Gibbons and Julie A. Hunt. Manuscript editing: Julie A. Hunt, Stanley Robertson and Stacy L. Anderson. Drafting of the manuscript: Stacy L. Anderson.

## Supporting information

Appendix 1 The student worksheet used to assess validity and reliability of journal articles

Appendix 2 Student survey

Appendix 3 The rubric used to grade the Fresno test

cohort.docx
