# Supplementary material for: Evaluation of an evidence‐based veterinary medicine exercise for instruction in clinical year of veterinary medicine program
Source: Vet Rec Open. 2021 Apr 2;8(1):e3. doi: 10.1002/vro2.3 (PMC8110120; doi:10.1002/vro2.3)
Supplement: Supplementary file 1 — Appendix 1 The student worksheet used to assess validity and reliability of journal articles [file VRO2-8-e3-s003.pdf]

<u>**Evidence Based Veterinary Medicine Exercise LA DVTC Rotation**</u>

Use the following form to help you assess your paper. Please turn this form in after the journal club session.

Clinical question:

Why did you choose this question?

PICO:

P-

I-

C-

O-

What type of question is this (e.g. treatment, prognosis)

Search Strategy

Databases searched and dates covered	
Search terms	
Exclusion criteria	
Inclusion criteria	

Search results

Database	Number of results	Number excluded	Reasons for exclusion	Total relevant papers

Summary of the Evidence

Reference (First author, year published)	
Population	
Sample size	
Intervention details	
Study design	
Outcome studied	

Main findings relevant to PICO question	
Limitations	

Full reference (Harvard reference style)

Appraisal, application and reflection (max. 400 words)
