# Supplementary material for: Evaluation of an evidence‐based veterinary medicine exercise for instruction in clinical year of veterinary medicine program
Source: Vet Rec Open. 2021 Apr 2;8(1):e3. doi: 10.1002/vro2.3 (PMC8110120; doi:10.1002/vro2.3)
Supplement: Supplementary file 2 — Appendix 2 Student survey [file VRO2-8-e3-s004.pdf]

Student # ____________

Evidence Based Medicine Questionnaire – pre exercise questionnaire

General

1. Age ______
2. Gender M / F / other
3. Class (graduation year) 2018 /2019/ 2020 / 2021
4. Years of undergraduate prior to veterinary school ___
5. Do you have an advanced degree Y / N
    a. If yes, please specify ___________________________
6. Have you completed a research project or participated in a research project as a researcher (not participant) Y/N

    If yes what role(s) did you have:

    a. Project design
    b. Data collection
    c. Statistical analysis
    d. Write up of paper
    e. Other (please describe)
7. How many clinical rotations have you had prior to this rotation? ____

Previous evidence based medicine courses

8. In one sentence please describe what you understand by ‘evidence based veterinary medicine’

9. Have you received any teaching on evidence based medicine previously? Y / N
    a. If no, proceed to question 10
    b. If yes,
        i. Was this undergraduate / graduate / professional (e.g. veterinary) school
        ii. Was this taught as lecture / laboratory / small group / other ______________ (circle all that apply)
        iii. How many hours did you receive? 1 / 2-5 / 5-10 / >10
        iv. Please feel free to add any comments

    c. Did this course involve any of the following (circle all that apply)
        i. How to search for an article
        ii. How to appraise an article
        iii. Statistics interpretation
        iv. Define PICO and development of PICO question

Student # _____________

10. Have you been asked to perform a search for literature by a clinician on a previous rotation
    a. If no, proceed to question 11
    b. If yes,
        i. How many times? 1 2-5 >6
        ii. What resources did you use (circle all that apply)
            1. Class notes/Blackboard resources
            2. Text book
            3. VIN
            4. General Google search
            5. Google Scholar search
            6. Pubmed, CAB abstracts or other database search for research papers
            7. Review articles
            8. Critically appraised topics
            9. Other (please specify)
        iii. How many minutes approximately did you dedicate to searching

            <10 11-30 31-60 >60

        iv. Were you able to download full peer reviewed articles

            Yes No

        v. Did you attempt to critically appraise the quality or validity of the information you obtained?

11. Have you undertaken a literature search to answer a clinical question based on a case you saw that the clinician did not ask you to perform i.e. this was self directed learning
    a. If no, proceed to question 12
    b. If yes,
        i. How many times? 1 2-5 >6
        ii. What resources did you use (circle all that apply)
            1. Class notes/Blackboard resources
            2. Text book
            3. VIN
            4. General Google search
            5. Google Scholar search
            6. Pubmed, CAB abstracts or other database search for research papers
            7. Review articles
            8. Critically appraised topics
            9. Other (please specify)

Student # _____________

iii. How many minutes approximately did you dedicate to searching

<10 11-30 31-60 >60

iv. Were you able to download full peer reviewed articles

Yes No

v. Did you attempt to critically appraise the quality or validity of the information you obtained?

12. Have you used the LMU Reed Medical Sciences Library to find articles and search databases Y/N

Likert scale questions

1. How confident are you in formulating a clinical question

Not at all confident	Slightly confident	Moderately confident	Quite Confident	Extremely confident
☐	☐	☐	☐	☐

2. How confident are you in your ability to find evidence

Not at all confident	Slightly confident	Moderately confident	Quite Confident	Extremely confident
☐	☐	☐	☐	☐

3. How comfortable are you with searching MedLINE and other databases

Not at all confident	Slightly confident	Moderately confident	Quite Confident	Extremely confident
☐	☐	☐	☐	☐

4. How confident are you in your ability to evaluate clinical evidence

Not at all confident	Slightly confident	Moderately confident	Quite Confident	Extremely confident
☐	☐	☐	☐	☐

5. How confident in your ability to use library resources to access the literature

Not at all confident	Slightly confident	Moderately confident	Quite Confident	Extremely confident
☐	☐	☐	☐	☐

Student # _____________

6. How likely are you to ask a librarian to assist you in finding information on a clinical topic

Almost never	Once in a while	Sometimes	Often	Almost always
☐	☐	☐	☐	☐

7. How confident are you in identifying an appropriate study type to answer your question

Not at all confident	Slightly confident	Moderately confident	Quite Confident	Extremely confident
☐	☐	☐	☐	☐

8. How important is the evidence based medicine to the practice of medicine

Not important	Slightly important	Moderately important	Quite important	Extremely important
☐	☐	☐	☐	☐

Student # _____________

Evidenced based veterinary medicine. Post exercise questionnaire

1. How useful was the EBVM seminar in improving your knowledge of EBVM

Not at all useful	Slightly useful	Moderately useful	Quite useful	Extremely useful
☐	☐	☐	☐	☐

2. How useful was the librarian seminar in improving your ability to search databases

Not at all useful	Slightly useful	Moderately useful	Quite useful	Extremely useful
☐	☐	☐	☐	☐

3. How useful was the librarian seminar in improving your ability to retrieve articles

Not at all useful	Slightly useful	Moderately useful	Quite useful	Extremely useful
☐	☐	☐	☐	☐

4. How useful was the journal club exercise in improving your knowledge of EBVM

Not at all useful	Slightly useful	Moderately useful	Quite useful	Extremely useful
☐	☐	☐	☐	☐

5. How useful was the FRESNO test in solidifying your knowledge of EBVM

Not at all useful	Slightly useful	Moderately useful	Quite useful	Extremely useful
☐	☐	☐	☐	☐

6. After completing this exercise how likely are you use knowledge learnt in future rotations

Not at all useful	Slightly useful	Moderately useful	Quite useful	Extremely useful
☐	☐	☐	☐	☐

7. Do you have any suggestions for improvement for the EBVM exercise?

Student # ____________

8. Any other comments

Evidence based veterinary medicine. Questionnaire at graduation

1. How many clinical rotations did you have after the EBVM exercise on the DVTC rotation? ____

2. How many times following the DVTC rotation did you develop a PICO question and perform EBVM on rotations (please circle)

   0 1-5 5-10 >10

3. Did you share the knowledge learnt in the EBVM exercise on the DVTC rotation with

   a. veterinarians at clinical affiliates Y / N
   b. veterinarians at practices you did an externship at? Y / N
   c. students from other institutions you encountered at the practice Y / N
   d. any other personnel? Please describe _______________________________________

The Institutional Review Board of Lincoln Memorial University (FWA00012543) has approved this research project #713 V.0 on June 21, 2018. This protocol will expire 365 days from approval unless renewed for another one (1) year period.
