# Supplementary material for: Evaluation of an evidence‐based veterinary medicine exercise for instruction in clinical year of veterinary medicine program
Source: Vet Rec Open. 2021 Apr 2;8(1):e3. doi: 10.1002/vro2.3 (PMC8110120; doi:10.1002/vro2.3)
Supplement: Supplementary file 3 — Appendix 3 The rubric used to grade the Fresno test [file VRO2-8-e3-s001.pdf]

DVTC Rotation Fresno test of Evidence Based Veterinary medicine RUBRIC

EBVM exercise 10% of grade. (150 points max)

100 points for FRESNO test

10 points for timely submission of of an appropriate clinical question

10 points for timely submission of an appropriate article

10 points for interaction and contribution to meaningful discussion in journal club

20 points for completion of the EBVM exercise sheet, scored based on rubric

Answer Questions based on the following clinical scenarios:

1. A stocker producer contacts you as he has a high incidence rate of respiratory disease in his high risk calves. Currently he uses oxytetracycline subcutaneously on arrival. He has been reading in Drovers Magazine about using 'newer' macrolide antibiotics instead of his current protocol or Draxxin that his neighbor uses.
2. A cow-calf producer contacts you to castrate and dehorn his calves. He asks if you will be using any local blocks because he doesn't think its worth the extra time and cost.

3. You diagnose many horses with distal tarsal joint osteoarthritis. Currently, you treat horses with intra-articular corticosteroids and judicial use of non-steroidal anti-inflammatory drugs. You keep seeing advertisements for new osteoarthritis modulating drugs, but have no experience using them.
4. A large horse boarding farm has been using a rotational anthelmintic program for the past 20 years. Lately, they have had an increase in the occurence of colic on their farm and would like you to evaluate their parasite control program.

1. A) Write a focused clinical question for ONE of the patient encounters that will help you organize a search of the clinical literature for an answer and chose the best article from among those you find

	Population	Intervention	Comparison	Outcome
Excellent (3)	Multiple relevant descriptors and appropriate combination of descriptors	Includes specific intervention of interest	Includes specific alternative of interest	Outcome that is objective and meaningful to search
Strong (2)	One appropriate descriptor	Mentions type of intervention	Mentions specific comparison group	Non specific outcome
Limited (1)	Single general descriptor unlikely to contribute to search e.g. patient	Mentions intervention but unlikely to contribute to search e.g. treatment	Mentions comparison but unlikely to contribute to search e.g. compared to other methods	Reference to outcome but so general as to be unlikely to contribute to search e.g. decrease disease
Not evident (0)	None	none		none

2. Where might clinicians go to find an answer to questions like these? Name as many possible types of categories of information sources as you can. You may feel some are better than others but discuss as many as you can to demonstrate your awareness of the strengths and weaknesses of common information sources in clinical practice. Describe the most important advantages and disadvantages for each type of information source you list.

	Variety of sources	Convenience	Clinical relevance	Validity
Excellent (6)	At least 4 types of sources listed including<br>• Electronic databases of original literature (Medline, CAB abstracts)<br>• Specific journals e.g. JAVMA<br>• Textbook	Discussion includes at least 2 specific issues of convenience or mentions the same issue which discussing 2 different sources.<br>Issues may include<br>• Cost<br>• Speed<br>• Ease of search<br>• Ease of use<br>• Availability	2 specific issues related to relevance, or mentions the same issue which discussing 2 different sources.<br>Issues may include<br>• Clinically relevant outcomes<br>• Written for clinical application<br>• Appropriate specialty focus	2 specific issues related to validity, or mentions the same issue which discussing 2 different sources.<br>Issues may include<br>• Certainty of validity e.g. quality is uncertain or needs to be

	• EBVM databases e.g. Bestbetsforvets<br>• People<br>• Medical website (e.g. VIN)<br>• General internet search<br>• Professional organization e.g. AABP, AAEP guidelines<br>• Consensus statements e.g. ACVIM<br>• Review articles e.g. ACVIM, VCNA		• Information applicable to patient e.g. location, species<br>• Includes specific interventions in questions<br>• Specificity<br>• Comprehensiveness of source	critically appraised)<br>• EBVM approach<br>• Expert bias<br>• Systematic approach<br>• Peer review<br>• Ability to verify<br>• Standard of care<br>• Enough information provided to critique validity<br>• Up to date/outdated
Strong (4)	3 types listed	1 specific issue	1 specific issue	1 specific issue
Poor (2)	2 sources listed	Mentions convenience but without explanation	Mentions relevance without explanation	Mentions validity without explanation
Not evidence (0)	Only 1 source	No mention	No mention	No mention

3. If you were to search Medline or CAB abstract for original research on one of these questions, describe what your search strategy would be. Bes as specific as you can about which topics and search categories (fields) you would search. Explain your rationale by taking this approach. Explain how you would limit your search if necessary and explain your reasoning.

	Search terms	Tags	Delimiters
Excellent (8)	3 or more terms that reflect PICO being considered	Description of search strategy reflects understanding that articles in databases are indexed by more than one field. Discusses one or more field/index/tag by name (MeSH,	Describers more than one approach to limiting search e.g. limit to beef cattle, or randomized controlled trials

		language, title word, pub title, etc) and provides rational for search strategy	
Strong (4)	2 PICO items	Names 1 or more field but doesn't provide plausible defense of search strategy	Describes only 1 common method of limiting search
Limited (3)	1 item from PICO	NA	NA
Not evident (0)		No evidence of understanding that articles tagged by different fields or indices	No valid techniques for limiting a search

4. What type of study design would be best able to address this question and why?

	Study Design	Justification
Excellent (12)	Names one of the best sources: RCT, systematic review/meta-analysis, randomized double blinded clinical trial	Includes well-reasoned justification that reflects understanding of the important of randomization and/or blinding. Explicitly connects randomization to reduction of confounding and/or blinding to observer or measurement bias
Strong (9)	Describers but does not call by name one of the best sources	Justification present but less clearly articulated
Limited (6)	Describes by name as less desirable study design e.g. cohort, prospective clinical trial, or longitudinal	Justification present and raises legitimate issues unrelated to randomization or blinding such as cost effectiveness ethical concerns or blinding.
Minimal (3)	Describers or names a poor study design e.g. case control, cross sectional, case report, retrospective	Attempted justification but arguments are nonspecific and do not demonstrate understanding, may mention blindness and randomization but without explanation
Not evident (0)		

5. When you find a report of original research on these questions, what characteristics of the study will you consider to determine if it is ***relevant***. Include examples

(Questions 5-7 address critical review of literature divided into relevance, validity, and magnitude of effect size. These may be arbitrary subdivisions of the process of critical review. Therefore respondents may describe issues of validity in answers to any of these 3 questions. Consider the responses to all 3 questions as one response when applying the criteria in the following rubric.

	The question	Description of subjects
Excellent (12 points)	Well-reasoned and thoughtful discussion of the relevance of the independent and dependent variables used in the study including examples/specific reasons<br>e.g. patient or disease oriented nature of problem<br>the congruence between the operational definition and the research question. E.g. is their measurement of the outcome realistic representation of the outcome we care about	Includes both:<br>• A clear expression of the importance of the link between the study subjects and target population.<br>• At least one example of a relevant disease or demographic characteristic<br>e.g. "were the patients similar to mine in terms of age and species or use?" "did patients have same level of disease severity as my patient?"
Strong (9)	Less thoughtful discussion of the relevance of the independent and dependent variables used in the study. May include specific concepts or examples without clear rationale. May refer to:<br>• the feasibility of the test or intervention<br>e.g. "is it feasible?" or "can I actually use it?"<br>• the patient or disease-oriented nature of the outcome<br>e.g. "look for patient-oriented outcomes" or "does the outcome matter to my patient?"	Includes one but not both:<br>• A clear expression of the importance of the link between the study subjects and target population. • At least one example of a relevant disease or demographic characteristic

	• the congruence between the operational definition and the research question e.g. "did they measure what they set out to Includes one but not both:<br>• A clear expression of the importance of the link between the study subjects and target population.<br>• At least one example of a relevant disease or demographic characteristic<br>e.g. "is the patient like mine?" or "education level of population"<br>study?"	
Limited (5)	Response implies consideration of how well the study addresses the question at hand, but offers little discussion about why this may be important<br>e.g. "what are the variables?"; "does it answer my question?"; "the outcome measure"; "the purpose of the study"; "will it impact my practice?";	Response implies consideration of the study subjects, but offers no discussion of the connection between study subjects and target population or specific characteristics of the sample<br>e.g. "is it an appropriate sample?" or "what was the response or participation rate?" or "what were the exclusion criteria?" or "selection bias" or "setting" or "where study was conducted"<br>Not
Not evident		

6. When you find a report of original research on these questions, what characteristics of the study will you consider to determine if its findings are valid? Include examples (You've already addressed relevance, and question 7 will ask how to determine the importance of the findings...for this question, focus on the validity of the study.)

(Questions 5-7 address critical review of literature divided into relevance, validity, and magnitude of effect size. These may be arbitrary subdivisions of the process of critical review. Therefore respondents may describe issues of validity in answers to any of these 3 questions. Consider the responses to all 3 questions as one response when applying the criteria in the following rubric.

	Internal validity
Excellent (24)	Lists or describes at least 5 issues important to internal validity, such as:<br>• Appropriateness of study design<br>• Adequacy of blinding<br>• Allocation concealment<br>• Randomization of group assignment<br>• Invalid or biased measurement ("followed own protocol?")<br>• Importance of comparison or control group<br>• Intention to treat analysis<br>• Consideration of appropriate covariates ("were other relevant factors considered?")<br>• Conclusions consistent with evidence ("do the results make sense?")<br>• Importance of follow-up of all study participants • Appropriate statistical analysis<br>• Sample size / Power<br>• Sponsorship<br>• When study was conducted<br>• Confirmation with other studies
Strong (18 points)	Identifies 3-4 specific issues as above.
Limited (10 points)	Identifies 2 specific issues as above.
Minimal (5 points)	Identifies one or mentions validity
Not evident	

7. When you find a report of original research on these questions, what characteristics of the findings will you consider to determine their magnitude and significance? Include examples. (You've already addressed relevance and validity…for this question, focus on how to determine the size and meaning of an effect reported in the study.)

(Questions 5-7 address critical review of literature divided into relevance, validity, and magnitude of effect size. These may be arbitrary subdivisions of the process of critical review. Therefore respondents may describe issues of magnitude and significance in answers to any of these 3 questions. Consider the responses to all 3 questions as one response when applying the criteria in the following rubric.)

	Magnitude	Statistical significance
Excellent (12 points)	Response must clearly discuss both:<br>• clinical significance ("what is the clinical significance?" or "how large a difference was found?")<br>• example(s) of effect size measurements (e.g., specificity, sensitivity, likelihood ratio of a test, number needed to treat, relative risk, absolute risk reduction, mean difference for continuous outcomes, positive or negative predictive value)	Well-reasoned and thoughtful discussion of the indices of statistical significance, including at least 2 specific examples of important related concepts such as:<br>• p-values • confidence intervals • power • precision of estimates • Type 1 or Type 2 error
Strong (9 points)	Response discusses one but not both:<br>• clinical significance ("what is the clinical significance?" or "how large a difference was found?") • example(s) of effect size measurements (e.g., specificity, sensitivity, likelihood ratio of a test, number needed to treat, relative risk, absolute risk reduction, mean difference for continuous outcomes, positive or negative predictive value)	Lists more than one concept (as above) with insufficient or absent discussion (e.g. "p-value and confidence intervals")<br>OR<br>Lists and discusses only one concept (e.g. "p-value less than <.05")
Limited (5 points)	Response only suggests consideration of clinical significance or size of effect.<br>(e.g. "does it matter?" "will it impact my practice")	Mentions need to assess statistical significance or names only one concept from above without further discussion (e.g. "p-values")
Not evident (0)		

Fresno test adapted from http://uthscsa.edu/gme/documents/PD%20Handbook/EBM%20Fresno%20Test%20grading%20rubric.pdf
